# Sesamin-mediated high expression of *BECN2* ameliorates cartilage endplate degeneration by reducing autophagy and inflammation

**DOI:** 10.18632/aging.205386

**Published:** 2024-01-26

**Authors:** Baining Zhang, Zhiwei He, Jialin Guo, Feng Li, Zhi Huang, Wenkai Zheng, Wenhua Xing, Manglai Li, Yong Zhu, Xuejun Yang

**Affiliations:** 1 Inner Mongolia Medical University, Hohhot, Inner Mongolia, China; 2 Department of Spine Surgery, Area A, The Second Affiliated Hospital of Inner Mongolia Medical University, Hohhot, Inner Mongolia, China; 3 Department of Bone and Soft Tissue Oncology, The Affiliated People’s Hospital of inner Mongolia Medical University, Peking University Cancer Hospital, Hohhot, Inner Mongolia, China

**Keywords:** Sesamin, BECN2, cartilage endplate degeneration, autophagy, inflammation

## Abstract

Lumbar disc degeneration (LDD) is a prevalent clinical spinal disease characterized by the calcification and degeneration of the cartilage endplate (CEP), which significantly reduces nutrient supply to the intervertebral disc. Traditional Chinese medicine offers a conservative and effective approach for treating LDD. We aimed to investigate the molecular mechanisms underlying the therapeutic effects of Sesamin in LDD treatment. Transcriptome sequencing was used to analyze the effect of Sesamin on LPS-induced ATDC5. We explored the role of BECN2, a target gene of Sesamin, in attenuating LPS-induced degeneration of ATDC5 cells. Our results revealed the identification of 117 differentially expressed genes (DEGs), with 54 up-regulated and 63 down-regulated genes. Notably, Sesamin significantly increased the expression of BECN2 in LPS-induced ATDC5 cell degeneration. Overexpressed BECN2 enhanced cell viability and inhibited cell apoptosis in LPS-induced ATDC5 cells, while BECN2 knockdown reduced cell viability and increased apoptosis. Furthermore, BECN2 played a crucial role in attenuating chondrocyte degeneration by modulating autophagy and inflammation. Specifically, BECN2 suppressed autophagy by reducing the expression of ATG14, VPS34, and GASP1, and alleviated the inflammatory response by decreasing the expression of inflammasome proteins NLRP3, NLRC4, NLRP1, and AIM2. *In vivo* experiments further supported the beneficial effects of Sesamin in mitigating LDD. This study provides novel insights into the potential molecular mechanism of Sesamin in treating LDD, highlighting its ability to mediate autophagy and inflammation inhibition via targeting the BECN2. This study provides a new therapeutic strategy for the treatment of LDD, as well as a potential molecular target for LDD.

## INTRODUCTION

Lumbar disc degeneration (LDD) is the primary contributor to low back pain and sciatica, placing a heavy burden on both individuals and society. The intervertebral disc is composed of the upper and lower cartilage endplates (CEP), the nucleus pulposus (NP), and the outer annulus fibrosus (AF) [1]. The nutrient and oxygen supply to the intervertebral disc is primarily facilitated by the penetration of CEP. Calcification and degeneration of CEP are the main causes of LDD as they reduce the supply of nutrients to the intervertebral disc [2]. Therefore, exploring the mechanism of CEP degeneration in relation to LDD is of significant importance.

The process of LDD involves the release of inflammatory cytokines, which can aggravate the degeneration of intervertebral disc [3]. Excessive destruction of the extracellular matrix has been clarified to be participated in the process of disc degeneration. Autophagy is a self-protection mechanism through which cells maintain homeostasis by breaking down damaged intracellular organelles in response to various adverse stimuli. Study has shown that autophagy exhibits a vital role in protecting the survival of NP and AF cells and delaying the degeneration of the intervertebral disc [4]. However, relatively few studies have focused on the mechanism of CEP degeneration.

At present, discectomy and non-operative treatment are commonly used in clinical trials to effectively alleviate the symptoms of LDD [5]. Because of several medical and surgical complications of discectomy, containing infection, nerve injury, symptomatic re-protrusion and secondary surgical injury, conservative treatment is considered to be a first-line option for most cases [6]. Traditional Chinese medicine is a conservative treatment approach for diseases, and it also has been demonstrated to effectively alleviate the symptoms of LDD [7]. Sesamin, a natural lignin health compound separated from sesame seeds, is generally used as a Chinese medicinal herb with various biological activities: anti-inflammatory [8], anti-oxidation [9], anti-cancer effects [10] and ameliorates rheumatoid arthritis [11]. Nevertheless, the key modulatory properties and characteristics of Sesamin, as well as its correlated mechanisms in attenuating CFP degeneration remain concealed.

In our study, transcriptome sequencing was performed to analyze the molecular mechanism of Sesamin in treating cartilage endplate degeneration. Moreover, we combined *in vivo* and *in vitro* LDD models to investigate the effects of Sesamin on a cartilage endplate degeneration model. We also explored the mechanisms underlying the anti-inflammatory and autophagy effects of Sesamin against the LDD pathological condition. Our study may provide a new treatment strategy for LDD.

## RESULTS

### Transcriptome analysis molecular mechanism of Sesamin on LPS-induced ATDC5 degeneration

**Figure 1:**
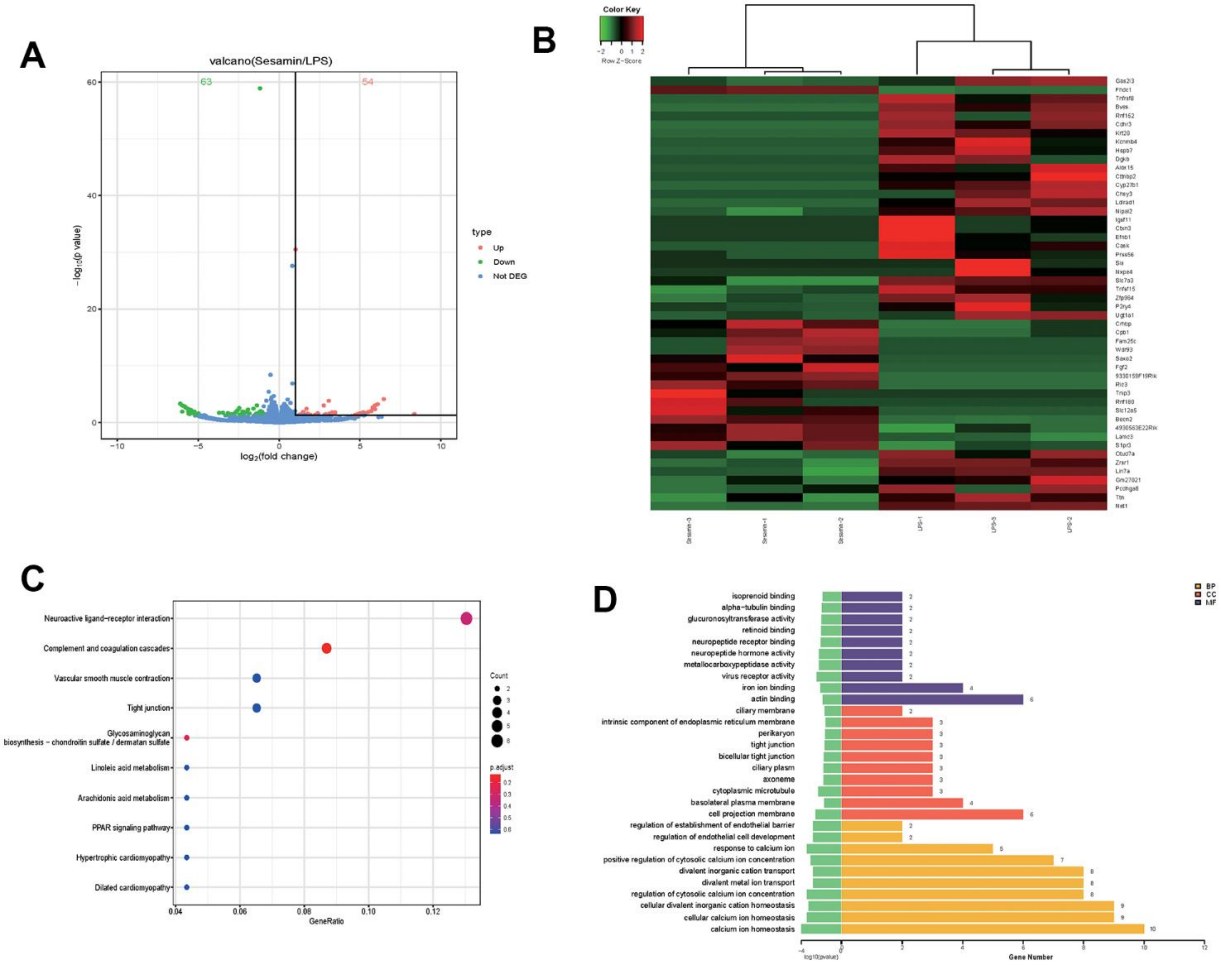
**Transcriptome analysis molecular mechanism of Sesamin on LPS-induced ATDC5 degeneration.** (**A**) volcano map of mRNA expression profiles. (**B**) The top50 of differentially expressed genes (DGEs). (**C**) The top 10 enriched Kyoto Encyclopedia of Genes and Genomes (KEGG) pathway of DEGs. (**D**) The top 10 enriched Gene Ontology (GO) pathway of the DEGs sorted by significance in biological process (BP), cellular component (CC) and molecular function (MF).

A total of 54 up-regulated and 63 down-regulated genes were identified using the cutoff criteria: *p*-values less than 0.05 and fold change greater than 2, as shown in the volcano map (Figure 1A). The top 50 DEGs were shown in the heat map (Figure 1B). Furthermore, Gene Ontology (GO) and Kyoto Encyclopedia of Genes and Genomes (KEGG) analyses were used to analyze the function of DEGs. In Figure 1C, the top 10 KEGG pathways of DGEs were mainly enriched in neuroactive ligand-receptor interaction, complement and coagulation cascades, vascular smooth muscle contraction, tight junction, glycosaminoglycan biosynthesis-chondroitin sulfate/dermatan sulfate, linoleic acid metabolism, arachidonic acid metabolism, PPAR signaling pathway, hypertrophic cardiomyopathy, and dilated cardiomyopathy. The results of the top 10 Gene Ontology (GO) pathways of DEGs in Figure 1D showed changes in biological processes (BP), cell component (CC) and molecular function (MF).

### *BECN2* overexpression decreased the apoptosis of LPS-induced ATDC5 cells by inhibiting autophagy and inflammation

**Figure 2:**
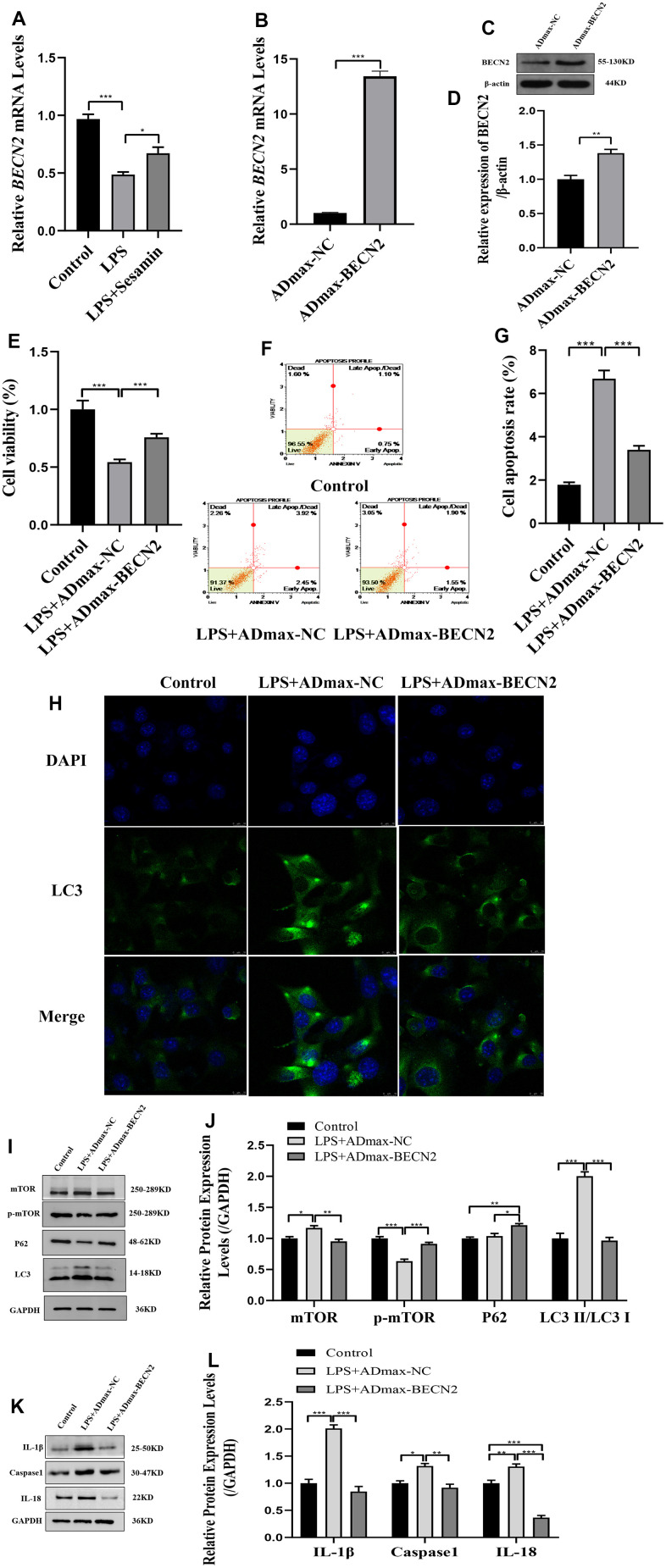
**The effect of BECN2 overexpression on the function of LPS-induced ATDC5 degeneration.** (**A**) The mRNA level of BECN2 was detected by RT-PCR in LPS-induced ATDC5 cells. (**B**) The effect of ADmax-BECN2 on the mRNA expression of BECN2 was detected by RT-PCR. (**C**) The protein level of BECN2 was evaluated by western blot. (**D**) The protein expression of BECN2 were determined using ImageJ software, GAPDH was used as the internal control, respectively (n=3). (**E**) CCK-8 was used to assess cell viability. (**F**, **G**) Cell apoptosis was detected by Muse. (**H**) The immunofluorescence staining of LC3 was used to evaluate the autophagy level (green signals represent LC3, blue signals represent DAPI, scale bar: 10 μm). (**I**, **J**) Autophagy-related protein expression levels were determined by immunoblotting. (**K**, **L**) The expression of IL-1β, Caspase1 and IL-18 were assessed by western blot. Relative protein expression was qualified by ImageJ software, GAPDH was used as the internal control, respectively. All data represent mean ± SD. All *in vitro* experiments were repeated three times independently. * *p* < 0.05, ** *p* < 0.01, and *** *p* < 0.001.

Based on transcriptome sequencing results, we found that BECN2 was significantly overexpressed in the Sesamin treated degeneration group, suggesting that Sesamin may participate in the degeneration of intervertebral cartilaginous endplate by regulating the BECN2 RT-PCR was used to verify the expression of BECN2, and the results in Figure 2A showed that Sesamin significantly increased BECN2 expression in LPS-induced ATDC5 cells (*p* = 0.042). ADmax-BECN2 adenovirus was used to overexpress the BECN2, and the overexpression efficiency was detected, as illustrated in Figure 2B–2D, indicating that ADmax-BECN2 significantly increased the expression of BECN2 in mRNA and protein levels (*p* < 0.001, *p* = 0.001, respectively).

We examined the effects of ADmax-BECN2 on cell viability, apoptosis and autophagy. And as shown in Figure 2F, 2G, the overexpression of BECN2 promoted cell viability (*p* < 0.001) (Figure 2E) and inhibited cell apoptosis (*p* < 0.001). The immunofluorescence results showed that ATDC5 cells formed fewer autophagosomes in the LPS+ADmax-BECN2 group than in the LPS+ADmax-NC group (Figure 2H). Moreover, the overexpression of BECN2 inhibited the expression of LC3II/LC3I and p-mTOR induced by LPS (*p* < 0.001, *p* < 0.001, respectively). However, the expression of P62 was increased in the LPS+ADmax-BECN2 group compared to the LPS+ADmax-NC group (*p* = 0.017) (Figure 2I, 2J). Additionally, the expression levels of inflammation related protein IL-1β, Caspase 1 and IL-18 were detected by immunoblotting, and the results indicated that LPS-induced high expression of inflammatory cytokines was inhibited by BECN2 overexpression (*p* < 0.001, *p* = 0.004, *p* < 0.001, respectively) (Figure 2K, 2L). Taken together, BECN2 overexpression could significantly inhibit apoptosis and autophagy induced by LPS in ATDC5 cells.

**Figure 3:**
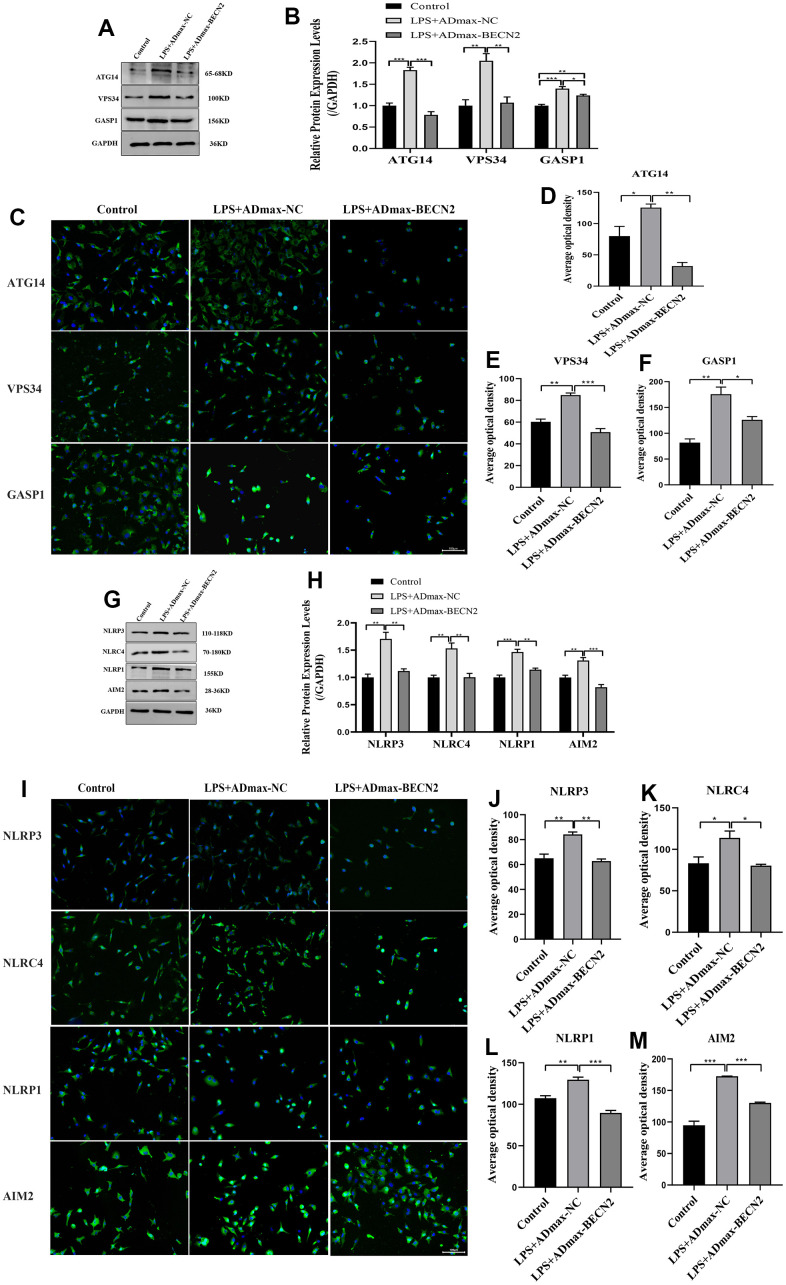
**Increased BECN2 attenuated the autophagy and inflammation of LPS-induced ATDC5 degeneration.** (**A**) Western blot was employed to detect the expression of ATG14, VPS34 and GASP1. (**B**) The protein expression of ATG14, VPS34 and GASP1 were determined using ImageJ software, GAPDH was used as the internal control, respectively (n=3). (**C**) ATG14, VPS34 and GASP1 expression were determined by immunofluorescence staining (scale bar: 100μm). (**D**–**F**) Average optical density was calculated by ImageJ software. (**G**) Western blot was employed to detect the expression of NLRP3, NLRC4, NLRP1 and AIM2. (**H**) The protein expression of NLRP3, NLRC4, NLRP1 and AIM2 were determined using ImageJ software, GAPDH was used as the internal control, respectively (n=3). (**I**) NLRP3, NLRC4, NLRP1 and AIM2 expression were determined by immunofluorescence staining (scale bar: 100μm). (**J**–**M**) Average optical density was calculated by ImageJ software. All data represent mean ± SD. All *in vitro* experiments were repeated three times independently. * *p* < 0.05, ** *p* < 0.01, and *** *p* < 0.001.

Furthermore, in order to explore the mechanism of BECN2 inhibiting LPS-induced ATDC5 degeneration, western-blot and immunofluorescence were used to detect the protein expression of autophagosome and inflammasome. The expression of autophagosome component proteins ATG14, VPS34 and GASP1 were showed in Figure 3A, 3B. Compared to LPS+ADmax-NC group, overexpressed BECN2 inhibited the expression of these proteins by a fold of 0.43,0.52 and 0.88 (*p* < 0.001, *p* = 0.008, and *p* = 0.045, respectively). The results of immunofluorescence in Figure 3C–3F were consistent with WB. Meanwhile, we observed that BECN2 overexpression significantly decreased the expression of NLRP3, NLRC4, NLRP1, and AIM2 by 0.65-fold (*p* = 0.005), 0.66-fold (*p* = 0.005), 0.77-fold (*p* = 0.004), and 0.63-fold (p = 0.004), respectively (Figure 3G, 3H). This result was verified by immunofluorescence, indicating that BECN2 may attenuate the degeneration of LPS-induced ATDC5 by inhibiting the formation of the inflammasome (Figure 3I–3M). These results indicated that BECN2 may protect chondrocyte degeneration by inhibiting autophagy and inflammation.

### *BECN2* knockdown increased the apoptosis of ATDC5 cells by promoting autophagy and inflammation

**Figure 4:**
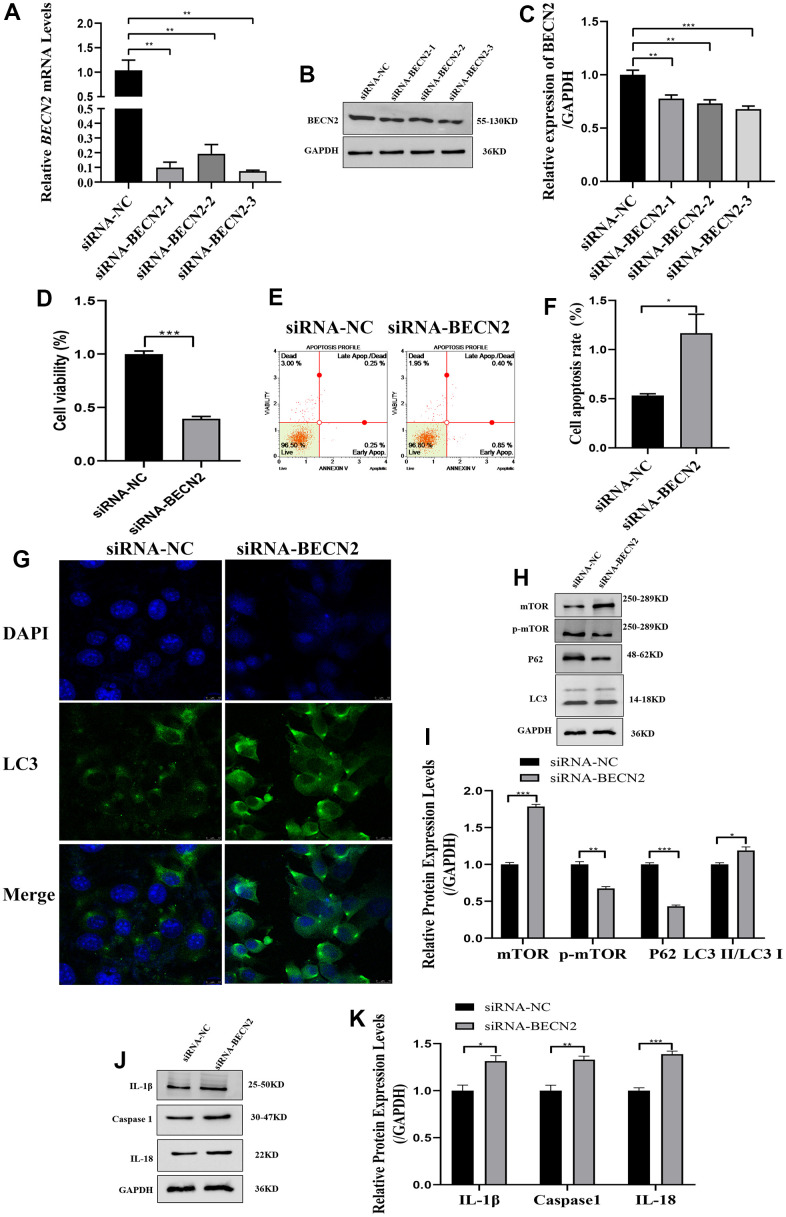
**The effect of BECN2 knockdown on the function of ATDC5.** (**A**) The effect of siRNA-BECN2 on the mRNA expression of BECN2 was detected by RT-PCR. (**B**) The protein level of BECN2 was evaluated by western blot. (**C**) The protein expression of BECN2 were determined using ImageJ software, GAPDH was used as the internal control, respectively (n=3). (**D**) CCK-8 was used to assess cell viability. (**E**, **F**) Cell apoptosis was detected by Muse. (**G**) The immunofluorescence staining of LC3 was used to evaluate the autophagy level (green signals represent LC3, blue signals represent DAPI, scale bar: 10 μm). (**H**, **I**) Autophagy-related protein expression levels were determined by immunoblotting. (**J**, **K**) The expression of IL-1β, Caspase1 and IL-18 were assessed by western blot. Relative protein expression was qualified by ImageJ software, GAPDH was used as the internal control, respectively. All data represent mean ± SD. All *in vitro* experiments were repeated three times independently. * *p* < 0.05, ** *p* < 0.01, and *** *p* < 0.001.

In order to further elucidate the role of BECN2 in inhibiting chondrocyte degeneration of Sesamin, we constructed the knockdown double-stranded siRNA. Interference efficiency was detected, and the optimal effect was observed with siRNA-BECN2-3 among the three interference sequences. The RT-PCR and WB results in Figure 4A–4C showed that siRNA-BECN2-3 decreased the expression of BECN2 mRNA and protein to levels 0.07-fold (*p* = 0.001) and 0.69-fold (*p* < 0.001) lower than that of siRNA-NC group. The results in Figure 4D showed that siRNA-BECN2 significantly decreased the cell viability to level 0.41-fold compared to siRNA-NC group. We noted that down-expressed BECN2 significantly promoted cell apoptosis, and the apoptosis rate reached 1.16% in Figure 4E, 4F (*p* = 0.031). The results were shown in immunofluorescence Figure 4G. Down-regulation of BECN2 increased the number of speckled autophagosomes. The results of autophagy related protein expression were shown in Figure 4H, 4I. The expression of p-mTOR and P62 were weakened by down-expressed BECN2 to levels 0.68-fold (*p* = 0.002) and 0.42-fold (*p* < 0.001) compared to siRNA-NC group. In contrast, the down-regulation of BECN2 resulted in an increase in the expression of LC3 II/LC3 I and total mTOR by 1.19-fold (*p* = 0.024) and 1.78-fold (*p* < 0.001), respectively. In addition, the expression of IL-1β, Caspase 1 and IL-18 were significantly increased induced by BECN2 knockdown to level 1.38-fold (*p* = 0.019), 1.31-fold (*p* = 0.009), and 1.39-fold (*p* < 0.001) in Figure 4J, 4K, respectively. These results suggested that BECN2 knockdown promoted the degeneration of ATDC5 chondrocytes.

**Figure 5:**
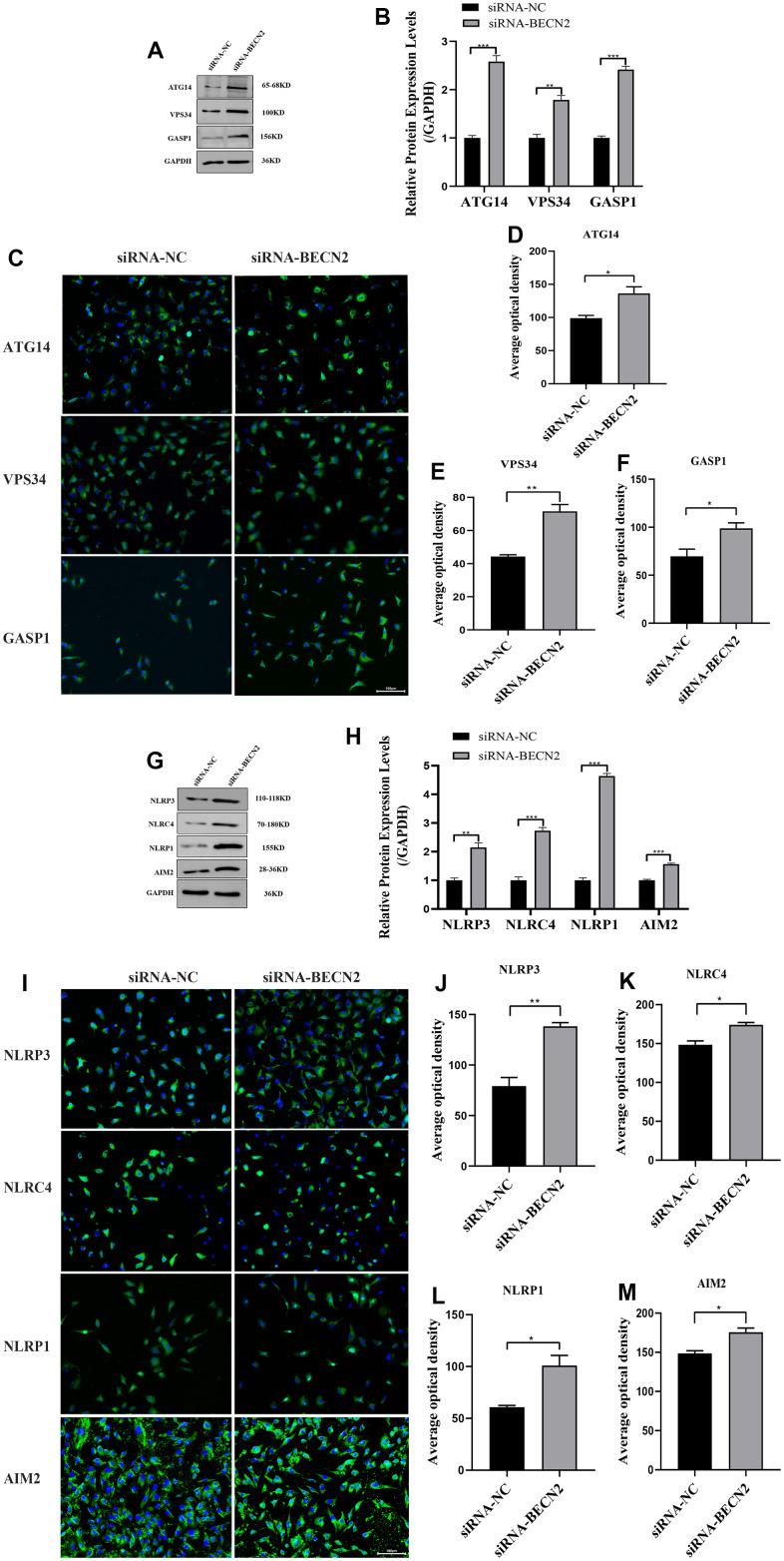
**Decreased BECN2 activated the autophagy and inflammation of ATDC5.** (**A**) Western blot was employed to detect the expression of ATG14, VPS34 and GASP1. (**B**) The protein expression of ATG14, VPS34 and GASP1 were determined using ImageJ software, GAPDH was used as the internal control, respectively (n=3). (**C**) ATG14, VPS34 and GASP1 expression were determined by immunofluorescence staining (scale bar: 100μm). (**D**–**F**) Average optical density was calculated by ImageJ software. (**G**) Western blot was employed to detect the expression of NLRP3, NLRC4, NLRP1 and AIM2. (**H**) The protein expression of NLRP3, NLRC4, NLRP1 and AIM2 were determined using ImageJ software, GAPDH was used as the internal control, respectively (n=3). (**I**) NLRP3, NLRC4, NLRP1 and AIM2 expression were determined by immunofluorescence staining (scale bar: 100μm). (**J**–**M**) Average optical density was calculated by ImageJ software. All data represent mean ± SD. All *in vitro* experiments were repeated three times independently. * *p* < 0.05, ** *p* < 0.01, and *** *p* < 0.001.

Furthermore, the western blot analysis in Figure 5A, 5B showed that ATG14, VPS34 and GASP1 expression were successfully promoted to levels 2.58-fold (*p* <0.001), 1.78-fold (*p* = 0.002) and 2.41-fold (*p* <0.001) by BECN2 siRNA. The immunofluorescence results in Figure 5C–5F showed that knockdown of BECN2 significantly increased the fluorescence signals of ATG14, VPS34, and GASP1 (*p* = 0.026, *p* = 0.003, *p* = 0.037, respectively). Similarly, BECN2 knockdown also increased the expression levels of NLRP3, NLRC4, AIM2, and NLRP1 by 2.14-fold (*p* = 0.003), 2.73-fold (*p* < 0.001), 1.56-fold (*p* < 0.001), and 4.64-fold (*p* < 0.001) respectively (Figure 5G, 5H). Subsequent immunofluorescence staining of NLRP3, NLRC4, NLRP1 and AIM2 showed that down-regulation of BECN2 by siRNA obviously promoted the formation of autophagosomes (*p* = 0.003, *p* = 0.011, *p* = 0.015, and *p* = 0.012, respectively) (Figure 5I–5M). These data indicated that BECN2 knockdown may promote chondrocyte degeneration by increasing autophagy and inflammation.

### Sesamin ameliorates LDD *in vivo*

**Figure 6:**
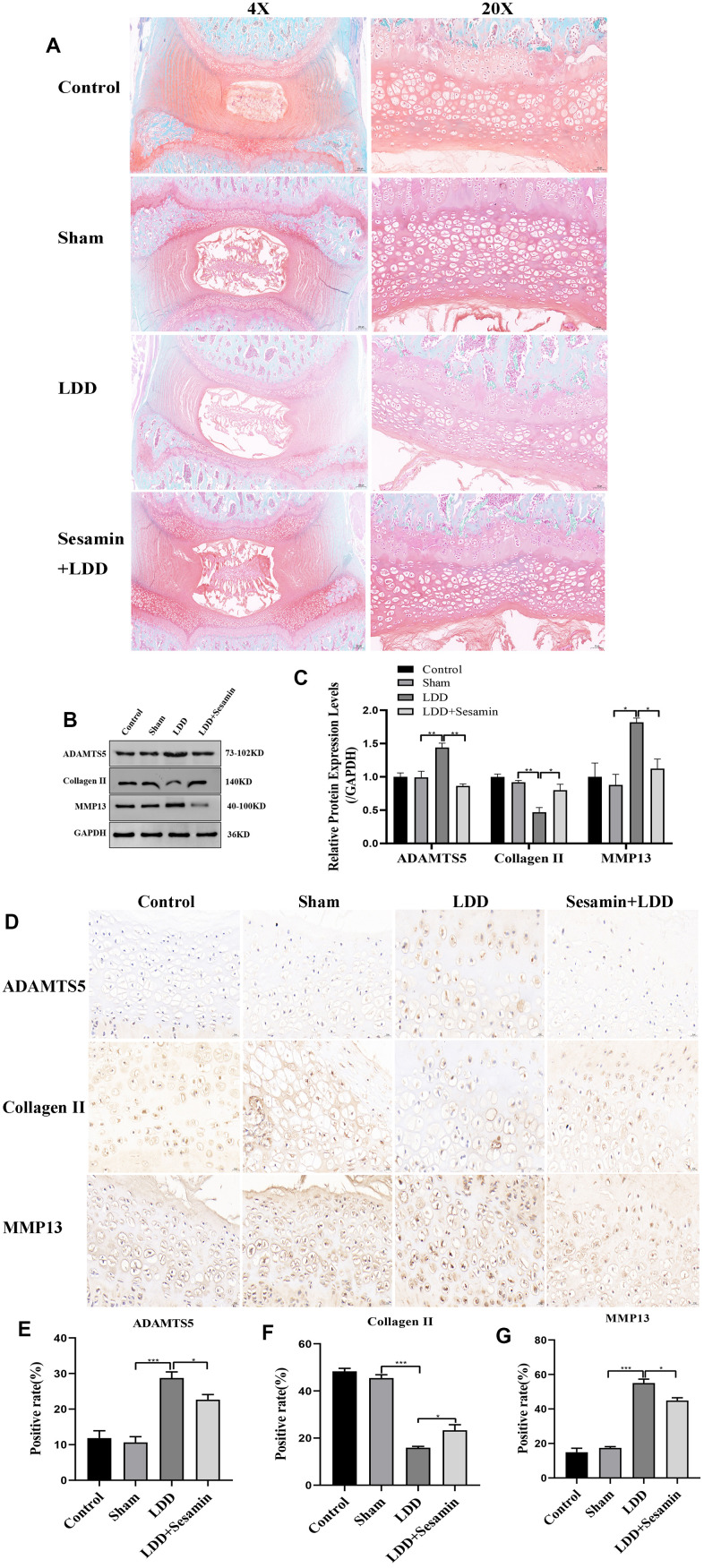
**Sesamin ameliorated degeneration of cartilage endplate in puncture-induced rat model.** (**A**) Representative images of the degenerative disc at 4 weeks post surgery by Safranin O-fast green staining (4X, scale bar: 200μm, 20X, scale bar: 5μm). (**B**) The protein level of ADAMTS5, Collagen II and MMP13 was evaluated by western blot. (**C**) The protein expression of ADAMTS5, Collagen II and MMP13 were determined using ImageJ software, GAPDH was used as the internal control, respectively (n=3). (**D**) ADAMTS5, Collagen II and MMP13 staining were detected (scale bar: 20 μm). (**E**–**G**) The positive rate was calculated by ImageJ software. All data represent mean ± SD. Three independent samples were used for statistics in all *in vivo* experiments. * *p* < 0.05, ** *p* < 0.01, and *** *p* < 0.001.

Moreover, the protective effect of Sesamin on cartilage endplate degeneration after 4 weeks of surgery was further confirmed by Safranin O-fast green staining, as shown in Figure 6A. Compared with sham group, we found that the number of cartilage cells was reduced, the staining degree was decreased, the cartilage cells were replaced by connective tissue, and cartilaginous endplate was collapsed in LDD group, while Sesamin treatment attenuated these degenerations. Meanwhile, cartilage endplate tissues were collected and examined the expression of ADAMTS5, Collagen II and MMP13 using RT-PCR, western blot and immunohistochemistry. The western blot results in Figure 1B, 1C showed significantly higher levels of ADAMTS5 (*p* < 0.001) and MMP13 (*p* < 0.001) in the puncture-induced rat model. However, under Sesamin treatment, the expression of ADAMTS5 (*p* = 0.011) and MMP13 (*p* = 0.036) was reduced. The protein level of Collagen II was obviously increased under Sesamin treatment compared to the LDD model (*p* = 0.019). The results were also confirmed by immunohistochemistry in Figure 1D–1G. These data suggested that Sesamin may alleviate the degeneration of rat cartilage endplate caused by acupuncture.

**Figure 7:**
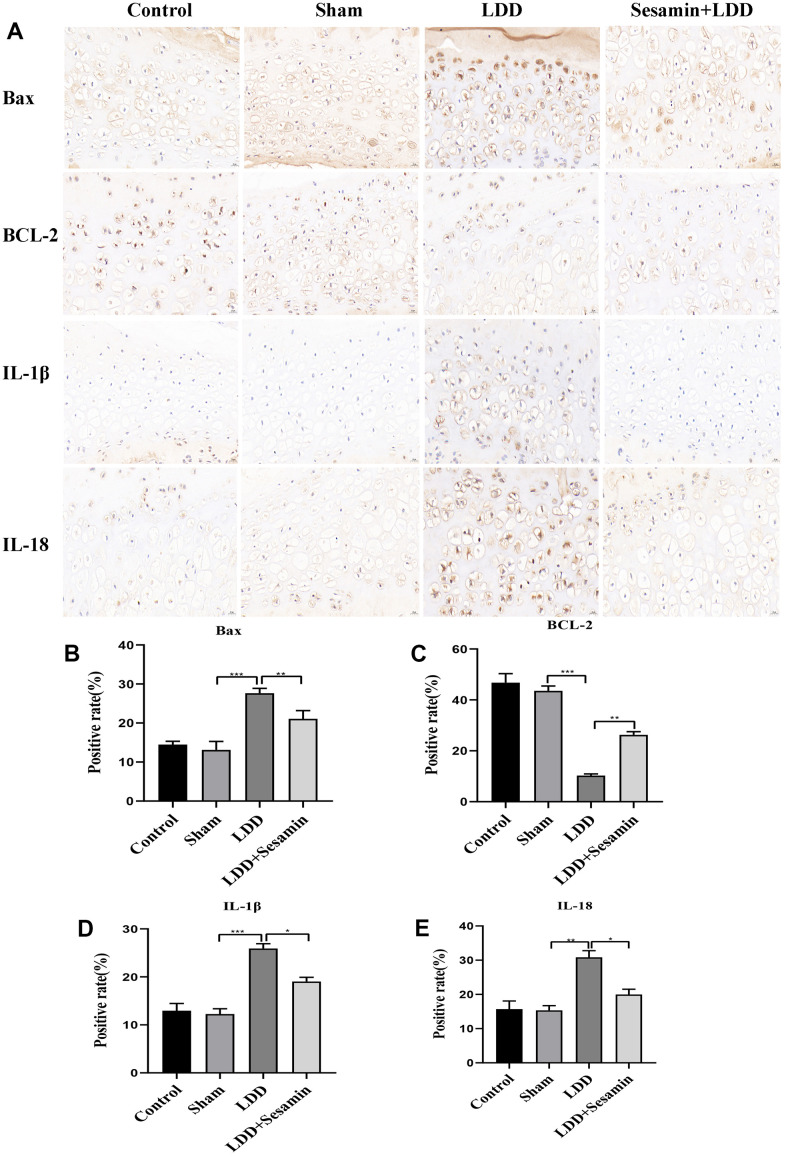
**Apoptosis was inhibited by Sesamin treatment in puncture-induced rat model.** (**A**) Bax, BCL-2, IL-1β and IL-18 were detected by immunohistochemistry (scale bar: 20 μm). (**B**–**E**) Relative positive rates were calculated by ImageJ software. All data represent mean ± SD. Three independent samples were used for statistics in all *in vivo* experiments. * *p* < 0.05, ** *p* < 0.01, and *** *p* < 0.001.

Additionally, immunohistochemistry was used to assess the expression of Bax, Bcl-2, IL-1β and IL-18 in Figure 7. In LDD model, the protein level of Bax was obviously promoted (*p* < 0.001), and Bcl-2 was inhibited (*p* < 0.001), these results were reversed by Sesamin treatment (*p* = 0.006, and *p* = 0.003, respectively) (Figure 7B, 7C). The results in Figure 7D, 7E showed that significantly higher positive rates of IL-1β (*p* < 0.001) and IL-18 (*p* = 0.002) were seen in the puncture-induced rat model, implying the activation of inflammation in LDD model group, however, Sesamin treatment significantly lowered the protein levels of IL-1β (*p* = 0.012) and IL-18 (*p* = 0.012). These results suggested that Sesamin regulated cartilage endplate degeneration through inflammation.

## Discussion and Conclusions

Lower back pain has been studied to be related to LDD, and by inference, it is associated with excessive manual, aging, and genetic factors. LDD not only brings a large deal of pain to personality but also causes large economic burden to society. Current studies have indicated that traditional Chinese medicine has a definitive impact on the conservative treatment of LDD and can facilitate the absorption of protrusions [12]. Sesamin is a lignan derived from sesame seeds. Research reports showed that Sesamin accelerated osteoporotic fracture healing via activating chondrogenesis and angiogenesis pathways [13], and another *in vitro* study reported that Sesamin prevented osteoporosis by promoting osteoblast differentiation [14]. In previous studies, we found that Sesamin could attenuate degenerative damage of LDD *in vitro* and *in vivo*. However, the underlying mechanism of Sesamin on LDD remains largely unknown. Hence, we investigated the mechanism of Sesamin alleviating LDD *in vitro*.

We explored for the first time the effect of Sesamin on the transcriptome of chondrocyte degeneration models, and 117 DEGs were found using cutoff criteria: *p*-values less than 0.05 and fold change greater than 2. We performed GO enrichment analysis and KEGG signaling pathway analysis for all the DEGs. We focused on BECN2 in the TOP50 gene. BECN2 mRNA expression level in the LPS-induced ATDC5 degeneration model was verified, and the result showed that Sesamin treatment significantly increased BECN2 expression. BECN2, a novel ATG6/Beclin family member, functions in an additional lysosomal degradation pathway via interacting with GASP1 as a ligand to initiate the lysosomal degradation pathway and thus participate in the regulation of body weight and glucose homeostasis [15]. Moreover, BECN2 was found to accelerate the endolysosomal degradation by interacting with herpesvirus, thus playing a role in innate immunity and control of viral oncogenesis [16].

In addition, the study showed that the expression of BECN2 was associated with the overall survival and disease-specific survival rates in patients with oral cancer, and the changes in BECN2 expression impacted the proliferation of the oral cancer cell line [17]. As far as we know, the role of BECN2 in LDD injury has not been reported. In our present study, we found that BECN2 overexpression alleviated the degeneration of LPS-induced chondrocyte via promoting cell viability and inhibiting apoptosis, while BECN2 knockdown decreased viability and increased apoptosis in the chondrocyte. These data indicated that BECN2 may represent a newly identified regulator of LDD.

Furthermore, we studied the molecular regulation of BECN2-induced apoptosis and found that BECN2 could significantly affect the autophagy of chondrocytes. Overexpressed BECN2 inhibited the formation of autophagosomes and increased the expression of p-mTOR and p62, while the expression of LC3 II/LC3 I decreased in the degeneration of LPS-induced ATDC5. Simultaneously, the autophagy-associated proteins ATG14, VASP34, and GASP1 were also inhibited in LPS-induced degeneration with BECN2 overexpression. And, we found that autophagy was promoted in ATDC5 with BECN2 knockdown. Autophagy is an intracellular catabolic process depends on the lysosome, involving the degradation of protein aggregates, organelles, and cytoplasmic protein. Pathologically enhanced autophagy is related to cell death, while it plays a protective role in most cases [18]. Autophagy has been found to be an essential pathological factor for the development of LDD [19], and a comprehensive analysis revealed that autophagy possesses bidirectional activity both inducing and inhibiting apoptosis in the LDD [20]. BECN2 is an autophagy-related protein and forms a complex with autophagy-inducing class III phosphatidylinositol 3-kinase (PtdIns 3-kinase) Vps34 inducing autophagy [21]. However, some researches have shown that the activation of autophagy in NP cells inhibited cell apoptosis and thus attenuated intervertebral disc degeneration [22], and according to another report, activating insulin-like growth factor1 signaling has been proved to antagonize the decreases in cell viability of human disc cells through enhancement of autophagy, thus inhibiting the development of LDD [19]. In our study, we found BECN2 inhibited ATDC5 apoptosis and attenuated LDD via reducing autophagy pathway. The autophagy mechanism of BECN2 under the special pathological conditions of LDD needs to be further verified.

Inflammation is a main factor responsible for the progression of lumbar disc herniation, and also a main reason for the low back pain [3]. Therefore, we explored the role of inflammation in BECN2-induced apoptosis inhibiting and LDD alleviating *in vitro*. The results showed that BECN2 overexpression decreased LPS-induced apoptosis of ATDC5, and the inflammatory factor caspase1, IL-1β, and IL-18 were all reduced. Moreover, the expression of inflammasomes NLRP3, NLRC4, NLRP1, and AIM2 were also inhibited. Inversely, BECN2 after knocking down, the expression of these proteins was rising, and the apoptosis of ATDC5 was promoted. Inflammasomes are multi-protein innate immune complexes that regulate Caspase-dependent inflammation and cell death. Increasing evidence has indicated that inflammasomes are associated with the initiation and progression of bone diseases [23]. During intervertebral disc degeneration, NLRP3 inflammasome is widely activated, which participates in regulating the production and release of key inflammatory cytokines, including IL-1 and IL-18 [24]. In the presence of danger signals or cellular events, AIM2 activates canonical inflammasomes, leading to the activation of Caspase 1, and secretion of IL-1β and IL-18 [25]. The facilitation of NLRP3 inflammasome activation and IL-1β releases promoted NP degeneration, and ultimately impacting on intervertebral disc degeneration [26]. At present, there are also some reports on the association between BECN2 and inflammation, and studies have reported that loss of Beclin 2 negatively regulates innate immune signaling and tumorigenesis by mediating the release of pro-inflammatory factors, and BECN2 is considered a potential target for the treatment of tumor and inflammatory diseases [27]. Moreover, the deletion of EBCN2 strengthened the activities of NLRP3, NLRC4, NLRP1, and AIM2, resulting in inflammation control, and it may serve as a target for the treatment of inflammatory diseases [28]. In our study, BECN2 negatively regulated the expression of inflammasomes, and thus participating in improving LDD.

In conclusion, this study provides novel insights into the potential molecular mechanism of Sesamin in treating LDD, highlighting its ability to mediate autophagy and inflammation inhibition via targeting the BECN2 gene for the treatment of cartilage endplate degeneration. This study provides a new therapeutic strategy for the treatment of LDD, as well as a potential molecular target for LDD. Further mechanistic experiments are needed to confirm our findings.

## MATERIALS AND METHODS

### Cell culture and cell injury model

The murine chondrogenic ATDC5 cells were obtained from the BeNa Culture Collection in Suzhou, China. Cells were cultured in DMEM/F12 medium (Hyclone, USA) supplemented with 10% (v/v) FBS (Hyclone, USA) and 1% (v/v) Penicillin-Streptomycin-Glutamine (Gibco, USA) in 50 cm^2^ cell culture flask. Flask was placed in a humidified incubator with 5% CO_2_ at 37° C. Lipopolysaccharide (LPS) was used to establish a cell injury model, and a concentration of 10 μg/ml LPS was selected for further experiments based on our previous research.

### Transcriptome sequencing

To understand the effect of Sesamin on the cartilage endplate degeneration, two groups were conducted, the LPS induced-ATDC5 group and LPS induced-ATDC5 treated with Sesamin (1 μmol/l) (MedChemExpress, USA) group. The cellular RNA was extracted for transcriptome analysis at Shanghai Genesky Biotechnology Company in Shanghai, China. The quality and quantity of the RNA used to generate the RNA sequencing libraries were evaluated using an Agilent Bioanalyzer 2100 system (Agilent Technologies, USA). The RNA library and RNA sequencing were performed using the Illumina HiSeq platform (Illumina, USA).

### Quantitative real-time polymerase chain reaction (qRT-PCR)

Total RNA was isolated from ATDC5 or cartilage endplate tissues using the TRIzol reagent (Invitrogen, USA). cDNA was synthesized from 1.0 μg of total RNA using the PrimeScript™ II 1^st^ Strand cDNA Synthesis Kit (TaKaRa, Japan). QRT-PCR was performed by the ABI 7500 Sequence Detection System (Applied Biosystems, USA). The relative gene expression, normalized to GAPDH, was calculated using the 2^-ΔΔCt^ formula. The primers used for qPCR are listed as follows. BECN2, Forward: 5’-TGCAACTAGACGACCAGCTC-3’, Reverse: 5’-CAGAGTACTCGACCCTGTGC-3’. GAPDH, Forward: 5’-CATCACTGCCACCCAGAAGACTG-3’, Reverse: 5’-ATGCCAGTGAGCTTCCCGTTCAG-3’.

### Cell transfection

For transient transfection, double-stranded siRNA oligonucleotides targeting BECN2 and negative control RNAs (siRNA-NC) were synthesized by Genepharma (Shanghai, China). ATDC5 cells were transfected with siRNA using Lipofectamine RNAiMax reagent (Invitrogen, USA). For adenovirus transfection, ADMax-NC and ADMax-BECN2 were constructed by the Shanghai Genepharma Company (Genepharma, China). The BECN2 interfering sequences were as follows: siRNA-BECN2-1: 5’- GAAGCUGACAGUCAGAACUTT-3’ (sense), 5’- AGUUCUGACUGUCAGCUUCTT-3’ (antisense), siRNA-BECN2-2: 5’- CCUGGCCAAUACAAUUGGATT-3’ (sense), 5’- UCCAAUUGUAUUGGCCAGGTT-3’ (antisense), siRNA-BECN2-3: 5’- CACUCAAGUUCAUGCUUAUTT-3’ (sense), 5’- AUAAGCAUGAACUUGAGAGUGTT-3’ (antisense). Negative control siRNA: 5’- UUCUCCGAACGUGUCACGUTT-3’ (sense), and 5’- ACGUCGACACGUUCGGAGAATT-3’ (antisense).

### Cell viability

For CCK-8 assays, treated cells were placed in quintuplicate in 96-well plates and cultured in DMEM/F12 medium containing 10% FBS for 24 h. Following the manufacturer’s instructions, the Cell Counting Kit-8 (MedChemExpress, USA) assay was introduced to measure cell viability at an optical density of 450nm.

### Cell apoptosis

Muse® Cell Analyzer (Millipore, USA) was adopted to assess the apoptosis in ATDC5 as per the manufacturer’s instructions. After trypsinization, the cells were harvested and subsequently washed twice with PBS. 100 μl of the Muse Annexin V and Dead Cell Reagent (Millipore, USA) was added to each sample, followed by incubation for 20 min at room temperature in the dark. The percentages of the four cell subpopulations were distinguished as follows: lower-left: viable cells, lower-right: cells in the early stages of apoptosis, upper-right: cells in the late stages of apoptosis, and upper-left: cells that have died via necrosis instead of apoptotic pathway.

### Western blotting

For total protein extraction, ATDC5 or cartilage endplate tissues from each group were lysed using RIPA lysis buffer (Beyotime, China). The proteins were separated by electrophoresis and transferred to a PVDF membrane (Millipore, USA). After being blocked with 5% skim milk for 1 h at room temperature, the blot was incubated with a specific primary antibody overnight. Subsequently, the HRP-conjugated secondary antibody was used to conjugate the specific primary antibody. ECL reaction images were acquired and observed using the Touch Imager XLI (e-BLOT, China). GAPDH or β-actin was adopted as an internal reference, and ImageJ v1.47 software (National Institutes of Health, USA) was applied to analyze the protein band images. The antibodies used for the western blot analysis are listed as follows. Anti-MMP13 (1:1000, 18165-1-AP, Proteintech), anti- MMP3 (1:1000, 17873-1-AP, Proteintech), anti-IL-1 Beta (1:1000, 16806-1-AP, Proteintech), anti--mTOR (1:1000, 66888-1-Ig, Proteintech), anti-LC3 (1:1000, 14600-1-AP, Proteintech), anti-P62/SQSTM1 (1:1000, 18420-1-AP, Proteintech), anti-Beclin 2 (1:500, NB110-60984SS, Novus), anti-NLRP3 (1:1000, 19771-1-AP, Proteintech), anti-ATG14 (1:1000, 19491-1-AP, Proteintech), anti-Phospho-NLRC4 (Ser533) (1:1000, AF3580, Affinity), anti-VPS34 (1:1000, 12452-1-Ap, Proteintech), anti-Caspase 1 (1:1000, 22915-1-AP, Proteintech), anti-IL-18 (1:1000, 10663-1-AP, Proteintech), anti-GPRASP1 (1:1000, 20020-1-AP, Proteintech), anti-NLRP1 (1:1000, DF13187, Affinity), anti-Phospho-mTOR (Ser2448) (1:1000, #5536, Cell Signaling Technology), anti-AIM2 (1:500, 122335, ZEN Bioscience), anti-GAPDH (1:10000, 60004-1-Ig, Proteintech), anti-ADAMTS5 (1:1000, DF13268, Affinity), anti-Collagen II (1:1000, Bs-0709R, Bioss) antibody. And secondary antibodies involved HRP-conjugated Affinipure Goat Anti-Rabbit IgG (1:20000, SA00001-2, Proteintech), and HRP-conjugated Affinipure Goat Anti-Mouse IgG (1:20000, SA00001-1, Proteintech).

### Immunofluorescence assay

ATDC5 cells for immunofluorescence staining were grown and handled on chamber slides. 4% formaldehyde and 0.2% Triton X-100 were used for cellular fixation and permeabilization. Subsequently, the cells were incubated with the specific primary antibody and fluorescently-labeled secondary antibodies. The immunofluorescence assay used the same specific primary antibody as the western blot, and the dilution ratio of the primary antibody was 1:50-1:200. The secondary antibodies are listed as below. Fluorescein (FITC) conjugated Affinipure Goat Anti-Mouse IgG (1:100, SA00003-1, Proteintech), Fluorescein (FITC)–conjugated Affinipure Goat Anti-Rabbit IgG (1:100, SA00003-1, Proteintech). The nuclei were stained with DAPI (Boster, China). Autophagosome were evaluated in the Leica TCS SP8 laser confocal fluorescence microscope (Leica, Germany), and the expression of proteins were observed by Nikon Ts2R inverted fluorescence microscope (Nikon, Japan). Fluorescence intensity was measured using ImageJ.

### Animals and establishment of rat LDD injury model

Forty-eight adult male Sprague-Dawley rats (200±20g, 6-8 weeks old) were randomly divided into four groups: the control group, the sham group, the LDD group, and the LDD + Sesamin group. All rats were anaesthetized with an intraperitoneal injection of 10% chloral hydrate (40 mg/Kg). Rat disc degeneration model was constructed by performing parallel punctures using a 0.4mm needle at the cartilaginous endplate for 30 sec. Three segments of the disc were selected for puncture in each rat, including L3/4, L4/5, and L5/6. Two weeks post-surgery, the rats in the LDD+Sesamin group were treated with 80mg/Kg Sesamin by intragastric administration for two weeks, and normal saline was used in the sham and LDD groups. In accordance with the standard of Rat Health Guide (http://ratguide.com/), the rats were monitored daily to guarantee their well-being. Four weeks after puncture, all rats were sacrificed, and the cartilaginous endplate tissue was used for the test.

### General histological and immunohistochemistry

Immunohistochemistry was performed to evaluate the expression and location of specific protein. Sections were incubated with primary antibodies against Bax (1:200, GB114122, Servicebio), IL-1β (1:100, GB11113, Servicebio), COL2 (1:200, GB11021, Servicebio), ADAMTS5 (1:100, TD13268M, Abcam), MMP13 (1:100, 18164-1-AP, Proteintech), Bcl-2 (1:100, 26593-AP, Proteintech), IL-18 (1:100, 10663-1-AP, Proteintech), and a secondary antibody. IHC was performed using the DAB solution and hematoxylin. Safranin-O/Fast Green staining is an approach used to assess the severity of cartilage injury. The Safranin-O/Fast Green staining kit (Servicebio, China) was utilized to stain and evaluate the distribution and composition of the extracellular cartilage matrix in the intervertebral disc.

### Statistical analysis

GraphPad Prism v.8.4.0 software was used to calculate the experimental data. All measurement data were shown as mean ± standard deviation. Differences were analyzed using Student’s t-test for comparing two groups and one-way analysis of variance for comparing multiple groups. All experimental data were obtained through independently repeated experiments for three times.
